# Improving the Phloroglucinolysis Protocol and Characterization of Sagrantino Wines Proanthocyanidins

**DOI:** 10.3390/molecules26041087

**Published:** 2021-02-19

**Authors:** Panagiotis Arapitsas, Daniele Perenzoni, Graziano Guella, Fulvio Mattivi

**Affiliations:** 1 Department of Food Quality and Nutrition, Research and Innovation Centre, Fondazione Edmund Mach, 38098 San Michele all’Adige, Italy; panagiotis.arapitsas@fmach.it (P.A.); daniele.perenzoni@fmach.it (D.P.); 2 Department of Physics, University of Trento, Via Sommarive 14, 38123 Povo Trento, Italy; graziano.guella@unitn.it; 3 Department of Cellular, Computational and Integrative Biology, CIBIO, University of Trento, 38098 San Michele all’Adige, Italy

**Keywords:** Sangiovese, Cabernet Sauvignon, Tannat, Merlot, Nebbiolo, polyphenols, tannins, condensed tannins, phloroglucinol, epicatechin, quantitative NMR

## Abstract

Proanthocyanidins are key metabolites that explain wine sensorial character (bitterness and astringency) and red wine color changes during aging. Therefore, a fast and accurate method to evaluate the degree of polymerization and the structural composition of the polymeric proanthocyanidins is a crucial analytical tool. Phloroglucinolysis is the most used method for this analysis but, unfortunately, the phloroglucinol adducts of the monomeric flavan-3-ols are not commercially available, making the results less accurate. The aim of this work was the isolation by semi-preparative high performance liquid chromatography (HPLC) of these non-commercial compounds and their use for the development of an accurate UHPLC-MS/MS protocol. The purity of each adduct was established via quantitative ^1^H-nuclear magnetic resonance (NMR) measurements with 3-trimethylsilyl-propionic-d4 acid sodium salt as the calibration standard. The developed method was applied to evaluate the proanthocyanidins profile of Sagrantino di Montefalco wines in comparison to other well-known tannic wines. Commercial, 6–8 years old Sagrantino wines were demonstrated to be very rich in epicatechin type B procyanidins, to have low galloylation %, and to have a high mean degree of polymerization of the proanthocyanidins with respect to the other analyzed wines.

## 1. Introduction

**Figure 1:**
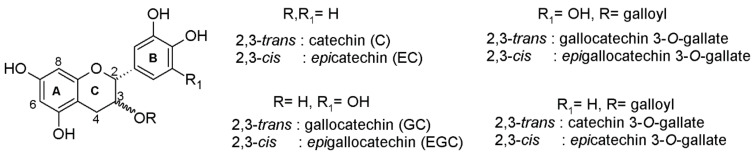
Flavan-3-ols basic structures.

Proanthocyanidins or condensed tannins is a group of secondary metabolites of paramount importance since they have a great impact on human health and food quality [1,2,3]. They can be found in large amounts in the majority of fruits, vegetables and plants, like wine, tea, berries, medicinal and aromatic plants and apples. Due to their quasi-ubiquitary presence in our plant food, the human average intake per day is very high in comparison with that of other classes of polyphenols [4,5]. In wine, proanthocyanidins play important roles, affecting color, astringency, bitterness, stability, aroma and aging potential [6,7,8,9,10]; and can be found in various forms, such as the monomeric catechin (C), epicatechin (EC), gallocatechin (GC), epigallocatechin (EGC) and epicatechin 3-gallate, their linear and cyclic oligomers/polymers, and their sulfonated adducts [6,7,11,12,13,14] (Figure 1). In wine science, there is a great demand for the structural elucidation and the absolute or relative quantification of the proanthocyanidins profile for various purposes like taxonomical aims, sensorial characterization, structure-function studies, biotic and abiotic stresses responses research and other technological issues [6,13,15,16,17].

Nowadays, with the development of the LC-DAD-MS instrumentation, the determination and quantification of the monomers and of the small oligomers is an easy target, through non-degradative methods. On the other hand, due to analytical and technological limitations, the direct analysis of the polymeric forms is a tricky and difficult task. A key limitation is due to the presence of several building units, which can be linked via the C4-C8 or C6-C8 bonds, which can theoretically generate several million potential combinations for an oligomer with a mean degree of polymerization (mDP) of nine or ten units, even without considering the additional presence of more complex structures, such as the type A proanthocyanidins and the cyclic (a.k.a. crown) proanthocyanidins. Therefore, during the last decades, many protocols have been developed to evaluate the structure and the mean degree of polymerization, by applying degradative methods. While the trapping reagent 2-mercaptoethanol has been widely used in the past, the protocols based on the acidic cleavage in the presence of phloroglucinol, followed by an LC-MS analysis are the most commonly used in the last years [7,18,19,20,21,22,23,24,25,26,27], especially when the desired target is the maximal conversion of proanthocyanidins into their constitutive subunit adducts [28]. The availability of pure standards of the flavan-3-ol phloroglucinol adducts are required for the quantification of acid-catalyzed phloroglucinol degradation products of procyanidins [29].

**Figure 2:**
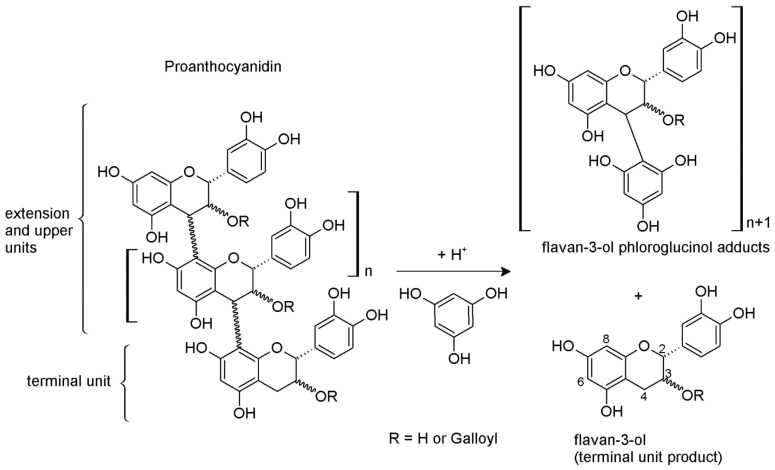
Phloroglucinolysis reaction.

According to this protocol (a.k.a. phloroglucinolysis), the interflavanoid bond of the polymeric proanthocyanidins is cleaved under acid conditions, releasing the terminal units as monomers and forming C4 phloroglucinol adducts of the extension units (Figure 2). Each sample is analyzed by chromatographic methods before (i.e., the blank) and after the reaction and the results of this assay, after subtraction for the blank, provides information about tannin structures, such as the mDP, the type and concentration of the terminal and upper sub-units and the average degree of galloylation [6,15,30,31]. The subtraction of the blank is required since the products of cleavage of the terminal units are the same flavan-3-ols already present in the wine. The use of the triple quadrupole mass spectrometer, as a detector connected to the liquid chromatography, allows the analyst to obtain the required results in a fast and efficient manner, including compounds present in trace amounts. However, this assay is not trivial. It must be done under accurately standardized conditions, preventing the possible degradation of the products of the reaction and especially of the terminal units. The comparison of the results in the literature obtained under different protocols is not straightforward. The yield of the assay depends on the reaction conditions. Moreover, some of the suggested applications omit to subtract the blank or do not mention this step in the materials and methods, and the phloroglucinol adducts of flavan-3-ols, which are the main reaction products, are sometimes quantified as equivalents of the free monomers by UV or MS detectors, due to the lack of commercial standards, thus providing less accurate results. Nevertheless, UV detectors permit us more accurate—but not absolute—quantification through calculation by using the bibliographic extinction coefficient values, but are less sensitive and require longer analysis time compared to MS detectors.

While the chemical role of the proanthocyanidins is shared by all the red wines, it has emerged that there is a huge diversity in the concentration of these compounds in the grapes from different cultivars [20,32,33,34]. It is expected that both the differences in the concentration, as well as in the structure, of the tannins can contribute to the huge diversity in the astringency of wines produced from them [35,36,37]. The need to build reliable reference values for the composition of each cultivar, to be used in the quality control, and combined with other data, especially the sensory data, calls for the need for accurate measurement via the phloroglucinolysis assay.

As an example of the application of this protocol, we chose to focus on Sagrantino wine. From this cultivar, it is produced a premium red Italian wine, the denomination of controlled and guaranteed origin (DOCG) Sagrantino di Montefalco, well-known for its rich tannic sensorial character [12,20,31,36], which can be considered a reference for the tannin-rich wines. Generally, the literature is lacking information in regard to the comparison of Sagrantino with other premium international wines with important tannic character.

The aim of this work was the preparation of flavan-3-ols phloroglucinol adducts in order to develop an LC-MS/MS method where their absolute quantification is possible. A secondary aim was to apply the method to several commercial red wines known for their tannic sensorial character and to compare them with Sagrantino commercial wines.

## 2. Results

### 2.1. Semi-Synthesis

**Figure 3:**
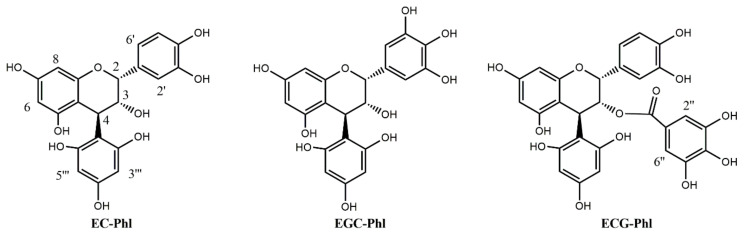
The three flavan-3-ol phloroglucinol adducts prepared, epicatechin 4-phloroglucinol (EP-Phl), epigallocatechin 4-phloroglucinol (EGC-Phl) and epicatechin-gallate 4-phloroglucinol (ECG-Phl).

The first part of this work was focused on the preparation of the flavan-3-ols phloroglucinol adducts. Initially, the phloroglucinolysis reaction was applied, using as starting material 10 g of a commercial apple extract rich in monomeric, oligomeric and polymeric flavan-3-ols such as catechin, epicatechin and type B procyanidin. Later, the use of a semi-preparative chromatography system allowed the isolation of epicatechin 4-phloroglucinol (EP-Phl) (Figure 3). With the same strategy and by using as starting material 30 g of a commercial grape seeds and skin tannins extract, it was possible to isolate both epicatechin 3-*O*-gallate 4-phloroglucinol (ECG-Phl) and epigallocatechin 4-phloroglucinol (EGC-Phl) (Figure 3). Another lot of these adducts was also prepared from the proanthocyanidins extracted from the skins of Zibibbo grapes. The structural confirmation was achieved by using nuclear magnetic resonance (NMR) and high-resolution MS instrumentation. ^1^H-NMR spectra of epicatechin 4-phloroglucinol (EC-Phl), epicatechin-gallate 4-phloroglucinol (ECG-Phl) and epigallocatechin 4-phloroglucinol (EGC-Phl) (reported in the Supplementary Figure S1) were used not only to confirm their structures (spectra taken in acetone-d6) but also to establish their concentrations through quantitative NMR procedures. In particular, we undertook these NMR measurements in D_2_O containing a known amount of internal standard (3-trimethylsilyl-propionic-d4-TSP) allowing us to establish, with good accuracy, the concentration of EC-Phl, ECG-Phl and EGC-Phl. These solutions were then used, as mother solutions, to prepare the corresponding working curves for LC-MS analysis. We suggest that quantitative ^1^H-NMR is a robust approach since it allows a direct measure of the absolute concentration present in the solution. With respect to the conventional measure of the extinction coefficients, this approach is not affected by the possible presence of traces of UV-absorbing impurities. Furthermore, there is no requirement to evaluate the variable presence of crystallization water, which is often a problem for highly hygroscopic compounds, such as the flavan-3-ols.

### 2.2. UHPLC-MS/MS Method

The developed method was based on previously described protocols [30,38] with small modifications to include the new metabolites. Table 1 shows the parameters of the LC-MS instrumentation for all the analyzed compounds, while Table 2 shows the parameters of the calibration curve.

### 2.3. Wine Analysis

In order to compare the amount and profile of the condensed tannins in Sagrantino wines with that of other red wines well-known for their tannic sensorial character, a total number of 133 commercial wines were included in the survey. These wines were produced between 2003 and 2012. In detail, 36 Sagrantino di Montefalco, 30 Sangiovese/Brunello di Montalcino, 17 Nebbiolo, 13 Cabernet Sauvignon, 13 Tannat, 12 Pinot noir, 4 Merlot, 3 Barbera, 2 Syrah, 2 Aglianico, and 1 Montepulciano were analyzed (Supplementary Table S1).

**Figure 4:**
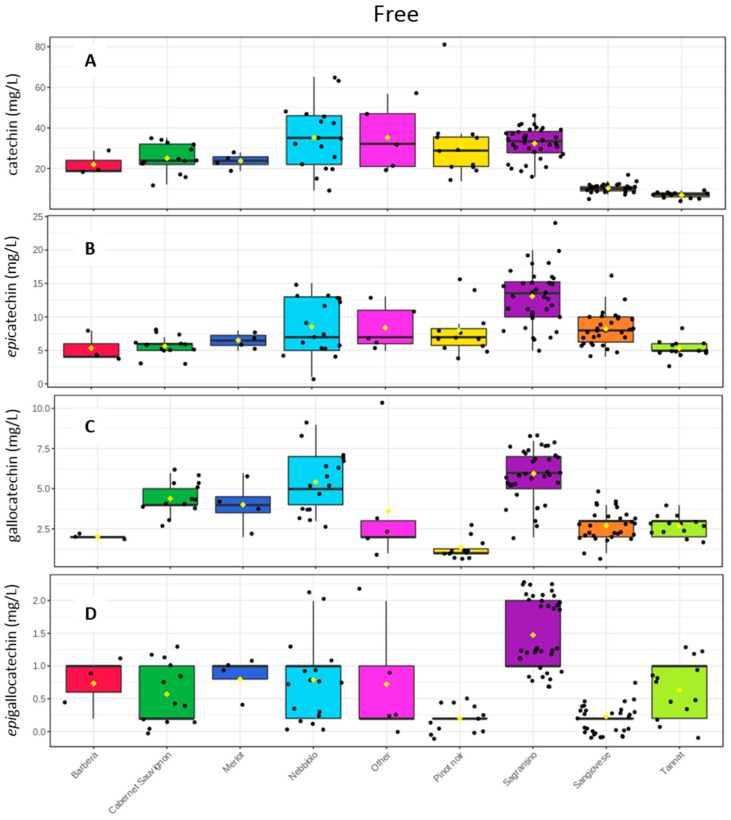
Box plots with the concentrations in monomeric flavan-3-ols before the phloroglucinolysis of (**A**) catechin, (**B**) epicatechin, (**C**) gallocatechin, and (**D**) epigallocatechin.

The use of the UPLC-MS/MS method before and after the phloroglucinolysis reaction demonstrated that, indeed, Sagrantino is a wine very rich in condensed tannins, with concentrations similar to, and sometimes higher than, the other analyzed wines. The full concentration data, together with the statistical analysis can be found in Supplementary Table S2. Figure 4 shows the distribution of four monomeric flavan-3-ols (catechin, *epi*catechin, gallocatechin and *epi*gallocatechin) before the reaction with phloroglucinol, of the different wine groups with box plots. Sagrantino wines were demonstrated to be rich in the *trans* forms of the flavan-3-ols, catechin and gallocatechin. In detail, they had a high amount of catechin (32 ± 7 mg/L), comparable to Merlot, Cabernet Sauvignon, Pinot noir and Nebbiolo (Figure 4A); and a high amount of gallocatechin, whose mean value of (6 ± 1.5 mg/L) was higher than Merlot, Cabernet Sauvignon and Nebbiolo wines, but this difference was not statistically significant (Figure 4C and Table S2). For the *cis* flavan-3-ols, *epi*catechin and *epi*gallocatechin, Sagrantino wines were shown the highest mean value with respect to the all other wine groups, since *epi*gallocatechin has a statistically significantly higher amount (1.4 ± 0.4 mg/L). Also *epi*catechin’s concentration was among the highest (1.4 ± 0.4 mg/L), together with Pinot noir and Nebbiolo (Figure 4B,D, and Supplementary Table S2). Moreover, for the dimeric procyanidin B1, Sagrantino wines have statistically significant the highest amount (62 ± 19 mg/L) and for procyanidin B2 was again among the highest (22 ± 8 mg/L), together with Pinot noir, Merlot, Cabernet Sauvignon and Nebbiolo (Supplementary Table S2).

**Figure 5:**
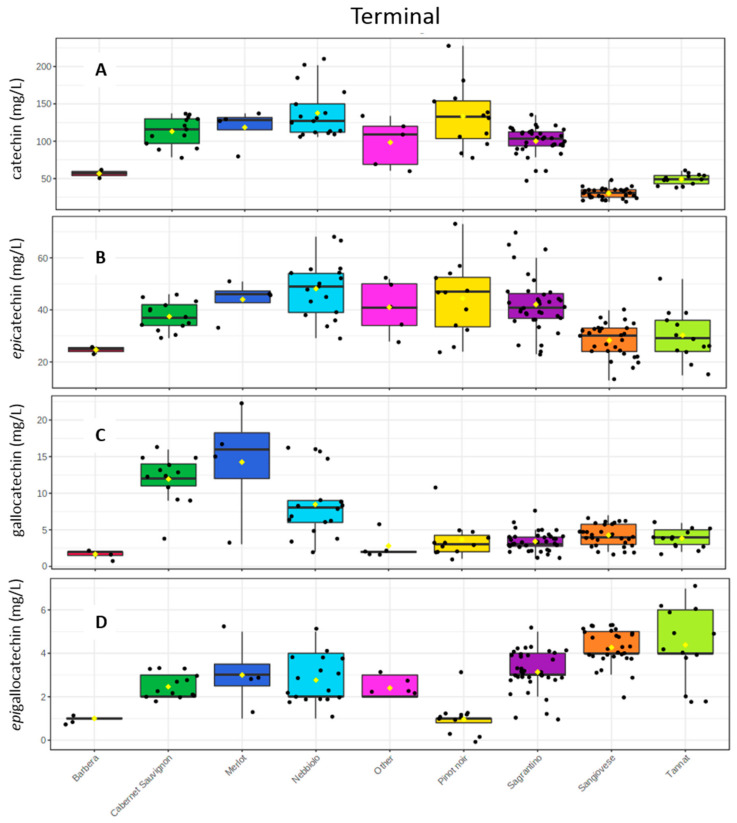
Box plots with the concentrations after the phloroglucinolysis of the “terminal” units of (**A**) catechin, (**B**) epicatechin, (**C**) gallocatechin, and (**D**) epigallocatechin.

The results obtained after the reaction with phloroglucinol confirmed the trend we registered previously, showing that Sagrantino is on average a wine rich in condensed tannins. The terminal units’ measurement demonstrated specifically that for catechin, *epi*catechin and *epi*gallocatechin, Sagrantino had among the highest amounts, with 100 mg/L (±19), 42 mg/L (±10) and 3 mg/L (±1) correspondingly. On the other hand, Sagrantino wines had low amounts of gallocatechin (3 ± 1 mg/L), with Merlot and Cabernet Sauvignon wines having the highest concentration among the cultivar investigated (Figure 5 and Supplementary Table S2).

**Figure 6:**
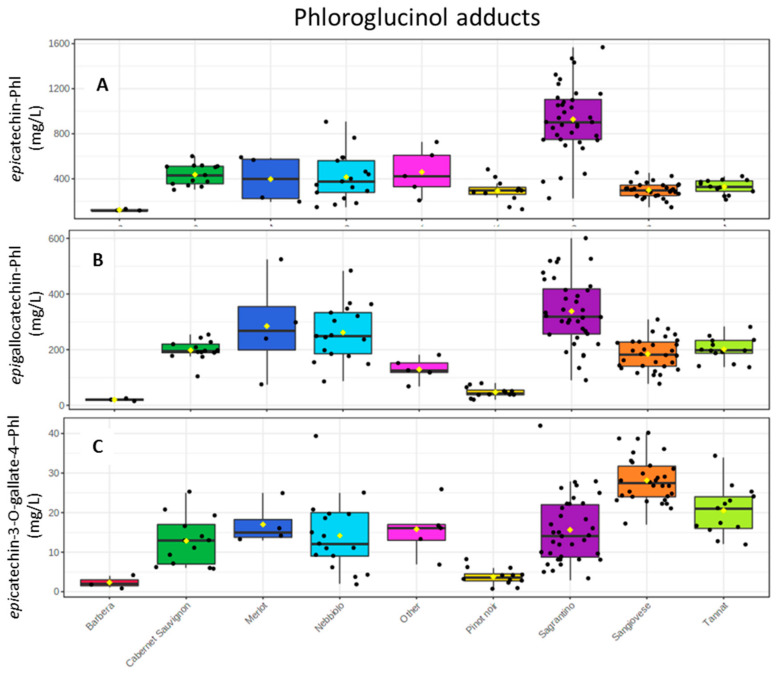
Box plots with the concentrations after the phloroglucinolysis of (**A**) epicatechin 4-phloroglucinol, (**B**) epigallocatechin 4-phloroglucinol, and (**C**) epicatechin 3-*O*-gallate 4-phloroglucinol.

The main products of the phloroglucinolysis are by far those derived from the extension and upper units of the oligomers, and the protocol applied allowed their accurate quantification against authentic standards. Figure 6 shows the comparison output of various wine groups according to their concentration in epicatechin 4-phloroglucinol, epigallocatechin 4-phloroglucinol and epicatechin 3-gallate 4-phloroglucinol. The most noticeable results were found for the epicatechin derivative since Sagrantino had by far the highest mean value, 928 mg/L (±303), which was more than double in respect to the second wine group, Cabernet Sauvignon (437 ± 95 mg/L). Sagrantino also had high amounts of epigallocatechin 4-phloroglucinol (338 ± 120 mg/L), but was still comparable to Cabernet Sauvignon, Merlot and Nebbiolo groups. On the contrary, Sagrantino had medium concentrations of epicatechin 3-gallate 4-phloroglucinol (16 ± 8 mg/L).

**Figure 7:**
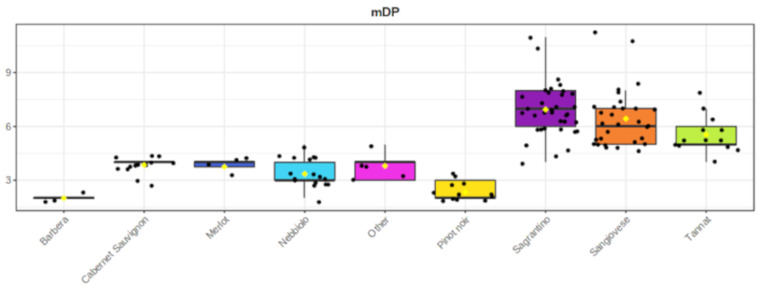
Box plots with the mean degree of polymerization (mDP) of the proanthocyanidins in the various wine groups. For the mDP calculation, the concentrations were expressed in mol.

Finally, Sagrantino wines had the highest mDP (6.9 ± 1) together with Sangiovese (5.6 ± 1) and Tannat (6.4 ± 1). The analyzed wines made from the international cultivars of Cabernet Sauvignon and Merlot had mDP 3.6 (±0.6) and 3.7 (±0.3) (Figure 7 and Supplementary Table S2).

## 3. Discussion

The first aim of this work was the development of a more accurate UHPLC-MS/MS protocol for the analysis of the polymeric proanthocyanidins after their depolymerization. According to the previous phologlucinolysis based protocols, the phloroglucinol adducts were quantified relatively as the free equivalents [21,30,31,39]. This problem, caused by the lack of commercially available standards, did not allow the correct multiple reaction monitoring (MRM) optimization; therefore, the researchers could not use the maximum sensitivity of the triple quadrupole mass spectrometer. This work described a semi-synthesis protocol that permits the preparation of the three most abundant flavanol 4-phloroglucinol derivatives, which were later used for the development of a fast and accurate UHPLC-MS/MS method for their quantification.

The prepared standards showed high stability through time since their methanolic solution stored at −20 °C for four years had a variation of their molar absorptivity lower than ±10%. The MRM optimization demonstrated that the phloroglucinol adducts had different optimum MS parameters (cone voltage and collision energies) with respect to their free analogs, indicating the importance of having them as pure standards for the method development. Moreover, the developed method had good linearity for all the analytes, with minimum of four orders of linearity and limit of detection (LOD) below 0.3 mg/L for all of them (Table 2). The sample preparation precision was below 20% for all the metabolites (≤10% for the majority) when the same pooled wine sample was prepared and analyzed 12 times. The inter-day and intra-day reproducibility were also good, with values comparable to the sample preparation variability. Generally, the analytical part of the protocol was in accordance with the literature outcome on the robustness and efficiency of the LC-MS/MS instruments in the analysis of the polyphenols/flavan-3-ols [18,21,22,23,40].

**Figure 8:**
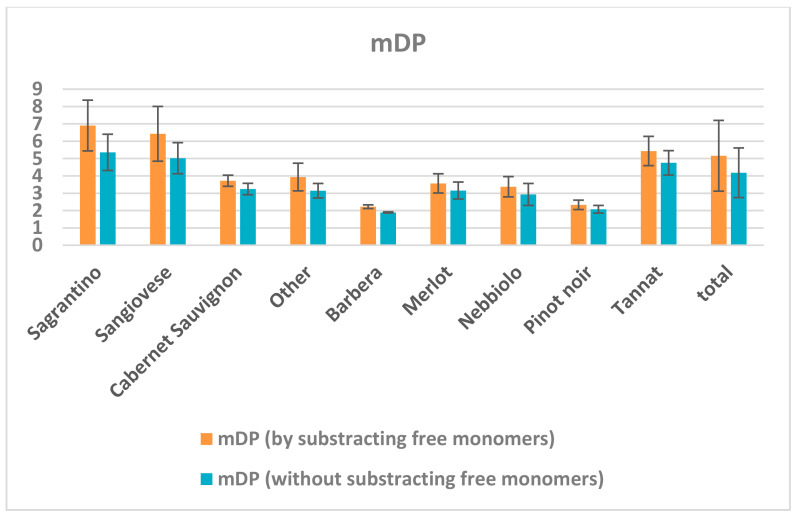
Comparison of the mDP values by subtracting or not the free monomeric flavan-3-ols concentrations before the phloroglucinolysis reaction. For the mDP calculation, the concentrations were expressed in mol/L.

Figure 8 demonstrates the systematic error whether we take into consideration the mDP calculation of the free monomeric flavan-3-ols concentration or not. For the calculation of free monomers, it is important to analyze each sample before the reaction with phloroglucinol, which doubles the time and cost of the instrumental (LC-MS) analysis, even if the sample preparation is far simpler in respect to the sample preparation for the measurement of the terminal units. In all samples, the calculated value was smaller when free monomers were not considered, with a loss between 6–40%. Nevertheless, all wines had similar age and winemaking protocols. For some wine cultivars the error was small, like Pinot noir (10.6 ± 4.5%) and Merlot (15.5 ± 0.3%), but for others it was relatively large, like Sagrantino (22.1 ± 2.5%) and Sangiovese (20.7 ± 6.2%). Apparently, wines with the higher mDP value had a higher error in terms of %, and not considering free units in the calculation could lead to poor accuracy of this estimate and wrong conclusions about their sensorial character.

Systematic errors are well known in analytical chemistry and not subtracting the blank sample (in our case the blank is the measurement of monomers before the phloroglucinolysis) can cause serious systematic bias. In the wine science literature on mDP calculation it is possible to find both protocols that subtract the free monomeric flavan-3-ols concentrations before the phloroglucinolysis reaction [24,25,30,41] and others that do not [19,20,21,22,23,26,27,28], while in some cases it is not clear which formula was used. This systematic error is probably small when comparing samples for the same cultivar and age, but it is better to avoid it when we compare samples from different cultivars or different ages.

A second critical issue, which can cause wrong considerations, could derive from the fact that this method does not consider the ethyl-bridged flavan-3-ols or the direct-linked and ethyl-bridged derivatives between anthocyanins and flavan-3-ols. For the ethyl-bridged flavan-3-ols, it was demonstrated that they represented less than 1.3% of the total interflavonoid flavan-3-ols in wine, therefore the error should be very small [19], while for the other adducts no information is available.

Knowing the length and the structural characteristics of the wine oligomeric and polymeric proanthocyanidins, also provides important information from the enological point of view. Condensed tannins participate in several reactions occurring during winemaking and wine aging, such as interflavan linkage formation and acidic cleavage, aldehyde polycondensation, sulfonation, oxidation, rearrangement and reaction with anthocyanin (direct and ethyl linked) [6,12,14,42,43,44]. Since the oligomeric and polymeric forms are the main source of this group of metabolites and technologically we are not yet able to measure the single molecules of the large forms with precision, a degradative protocol is still the best tool for obtaining information about these high molecular weight compounds. Wines with high mDP should be more predisposed to favoring the above reactions for a longer time, and winemakers should be aware of this feature. According to our results, Sagrantino wines belong to this group of wines.

Finally, our results could somehow explain the “dry,” “harsh” and “dynamic” astringent character, as well as the bitterness of Sagrantino wines [36,37]. The mDP value is only one of the parameters that is able to explain the influence of proanthocyanidins in wine sensorial character. Kallithraka et al. demonstrated that, for the same concentration, epicatechin is more astringent and especially more bitter than catechin [45]. According to Vidal et al. (2003) “drying” astringent character is positively correlated with mDP and degree of galloylation, while epigallocatechin units tend to lower the “coarse” perception [46]. In the light of these results, and by comparing the 133 commercial wines of this study, the Sagrantino wines’ astringent character could be explained with the following four parameters:

- The high mDP values have an impact on its “dry” astringent character.
- The high epicatechin amounts (and monomeric flavan-3-ols in general) should be correlated with its astringent and bitter character.
- The high oligomeric procyanidins concentration (Sagrantino wines have the highest mean value with 1.2 g/L and the second rich group is Nebbiolo with 0.8 g/L. (Table S2)), which should also explain the strong tannic character.
- The low % of galloylated proanthocyanidins do not allow the decrease of the “coarse” perception.

Of course, these are general hypotheses that require specific experiments for their validation, since the explanation of the sensorial character of a wine is dependent on several composition parameters.

## 4. Materials and Methods

### 4.1. Wine Samples

The sample list included 133 commercial red wines produced between 2003 and 2012, and was made up of 36 Sagrantino di Montefalco (Italy, 2004–2006), 30 Sangiovese/Brunello di Montalcino (Italy, 2004–2006), 17 Nebbiolo (Italy, 2004–2006), 13 Cabernet Sauvignon (Italy, 2004–2007), 13 Tannat (Uruguay, 2003–2012), 12 Pinot noir (Italy and France, 2004–2006), 4 Merlot (Italy, 2004–2006), 3 Barbera (Italy, 2004–2006), 2 Syrah (Italy, 2004–2005), 2 Aglianico (Italy, 2006), and 1 Montepulciano (Italy, 2006). The full list of the wines with their relative meta-information can be found in Supplementary Table S1. Blended wine was assigned to a group according to cultivar with the highest percentage.

### 4.2. Chemicals

Phloroglucinol and all the solvents for the reactions, extraction, isolation and analysis were purchased from FLUKA Sigma-Aldrich (St. Louis, MO, USA). Highest purity grade (+)-catechin, (−)-epicatechin (EC), (−)-gallocatechin (GC), (−)-epigallocatechin (EGC), (−)-epicatechin gallate (ECG), procyanidin B1 and procyanidin B2 were obtained from TransMIT PlantMetaChem (Gießen, Germany). Water (H_2_O) purified by a Milli-Q water purification system was used for all purposes.

### 4.3. Epicatechin 4-Phloroglucinol

For the preparation of epicatechin-(4→2)-phloroglucinol, a commercial apple extract (Pfannen Schmidt, Hamburg, Germany. Art-Nr: 11278112; Batch: 1005013-21) was used, which contained 12 mg/g of catechin, 29 mg/g of epicatechin and 53 mg/g of type B procyanidins. In 500 mL of 0.1N methanolic HCl (0.84 mL 37% aqueous HCl in 100 mL methanol) 10 g of the commercial apple tannin and 30 g of phloroglucinol were dissolved and stirred in a water bath at 50 °C for 1 h. Then the reaction was stopped by adding 100 mL of 0.4 N acetate sodium. Methanol was removed using a rotary evaporator and after 150 mL of H2O was added to the residue. The first step of purification was made using an Isolute C18 20g/70 mL Biotage (Uppsala, Sweden) cartridge. Once the cartridge was activated with 20 mL of methanol and conditioned with 40 mL of H_2_O, all the 150 mL of the above reaction solution was loaded and afterwards the cartridge was washed with 50 mL water, and finally the fraction with epicatechin-(4→2)-phloroglucinol was eluted with 100 mL of 10% aqueous methanol. This last fraction, after evaporation to dryness, was dissolved into in 3 mL of H_2_O and used for the HPLC semi-preparative isolation. A Waters 2695 HPLC equipped with a Waters 996 DAD detector (Waters Corp., Milford, MA, USA) and a Supelco Discovery C18 (5 μm, 4.6 × 250 mm a column) were used for the isolation. The flow rate was 1.5 mL/min, the eluents were water (A) and acetonitrile 0.5% acetic acid (B), the injection volume was 100 μL, and the column oven temperature was 40 °C. The gradient was isocratic (90% A) for the first 7 min, from 7 to 11 min isocratic with 100% B, and finally isocratic again with 90% A from 11 to 16 min. Automatic fractionation was carried out using a Fraction Collector III (Waters). The purity was calculated based on the quantitative ^1^H-NMR analysis described in Section 4.6.

### 4.4. Epicatechin 3-O-Gallate 4-Phloroglucinol

For the preparation of these two flavanol-phloroglucinol derivatives a commercial grape seeds and skin tannins extract (Enologica Vason S.p.A., Corrubbio, Italy) was used. In 1000 mL of 0.1 N methanolic HCl (0.84 mL 37% HCl in 100 mL methanol) 30 g of the commercial grape seeds and skin tannins and 90 g of phloroglucinol were dissolved and stirred in a water bath at 50 °C for 1 h. Then the reaction was stopped by adding 300 mL of 0.4 N acetate sodium. Methanol was removed using a rotary evaporator and after 150 mL of H20 was added to the residue. The first step of purification was made using an Isolute C18 20 g/70 mL Biotage (Uppsala, Sweden) cartridge. Once the cartridge was activated with 20 mL of methanol and conditioned with 40 mL of H_2_O, all the 150 mL of the above reaction solution was loaded, afterwards the cartridge was washed with 50 mL water, and finally the fraction rich in epicatechin-gallate-(4→2)-phloroglucinol was eluted with 100 mL of 5% aqueous methanol. This last fraction, after evaporation to dryness, was dissolved into 3 mL of H_2_O and used for the HPLC semi-preparative isolation. The isolation was achieved with the Waters 2695 HPLC set up described above. The gradient used was an isocratic at 88% A. The purity was calculated based on the NMR analysis described at Section 4.6.

### 4.5. Epigallocatechin 4-Phloroglucinol

For the preparation of this flavanol-phloroglucinol derivative, Zibibbo grape berries grown in Pantelleria (Italy) were used as a primary source. Only the skins from 3 kg of these berries were extracted with 3 L of methanol at room temperature and in the dark. To minimize oxidation, 1 g of ascorbic acid was added to the methanolic solution, which was also sparged with nitrogen [47]. After 24 h, the extract was concentrated under reduced pressure at 35 °C to remove methanol. To 1000 mL of this methanolic extraction we added 8.4 mL of 37% HCl and 30 g of phloroglucinol, and the reaction solution was stirred in a water bath at 50 °C for 1 h. Then the reaction was stopped by adding 300 mL of 0.4 N acetate sodium. Methanol was removed using a rotary evaporator and after 150 mL of H_2_0 was added to the residue. The first step of purification was made using an Isolute C18 20 g/70 mL Biotage (Uppsala, Sweden) cartridge. Once the cartridge was activated with 20 mL of methanol and conditioned with 40 mL of H_2_O, all the 150 mL of above reaction solution were loaded, afterwards the cartridge was washed with 50 mL water and finally the fraction rich in epigallocatechin-(4→2)-phloroglucinol was eluted with 100 mL of 5% aqueous methanol. This fraction, after evaporation to dryness, was dissolved into in 10 mL of H_2_O and used for the HPLC semi-preparative isolation. 

A Waters 2695 HPLC equipped with a Waters 996 DAD detector (Waters Corp., Milford, MA, USA) and a Supelco Discovery HS C18 (10 μm, 10 × 250 mm a column) were used for the isolation. The flow rate was 2.5 mL/min, the eluents were water 0.1 trifluoroacetic acid (A) and methanol (B), the injection volume was 70 μL, and the column oven temperature was 40 °C. The gradient was isocratic (91% A) for the first 13 min, from 13 to 17 min isocratic with 100% B, and finally isocratic again with 91% A from 17 to 21 min. Automatic fractionation was carried out using a Fraction Collector III (Waters). The purity was calculated based on the NMR analysis described at Section 4.6.

### 4.6. Quantitative ^1^H-NMR Spectroscopy

Acquisition: One-dimensional ^1^H-NMR spectra were acquired at 300 K on a Bruker Avance spectrometer (Bruker Biospin, Karlsruhe, Germany) equipped with a broadband inverse (BBI) probe and operating at a proton frequency 400.13 MHz. A single pulse sequence (zg) with 60° flip angle, without water pre-saturation, was used. All ^1^H-NMR measurements were carried out with a spectral width of 11 ppm (−1 to 10 ppm), a relaxation delay of 10 s, acquisition time of 7.5 s, 256 transients collected with 64k data points following four dummy scans without sample spinning. Samples were prepared by the addition of 0.750 mL of D_2_O containing 0.05% *w*/*w* of 3-trimethylsilyl- propionic-2,2,3,3-d4 acid sodium salt (TSP). TSP was used both as a reference standard (δ_H_ = 0.00 ppm) and as a calibration standard (C_(STP)_ = 3.2 mM). In order to validate the TSP concentration in the D_2_O vials used to prepare the NMR samples of EC-PhL, EGC-PhL and ECG-PhL, it was titrated by adding a known amount of pure (<99%) tryptophan (Trp) to the NMR tube containing 0.72 mL of D_2_O, which contained 0.05% *w*/*w* of TSP. The TSP concentration was determined as C_(TSP)_ = 3.20 ± 0.02 mM by the relative area integration of the TSP and Trp signals.

Spectral Processing: Spectra were analyzed both by TopSpin 3.5 (Bruker BioSpin) and by MestReNova 14.1.2 software package (Mestrelab Research S.L., Santiago de Compostela, Spain). Before Fourier transformation, all spectra were apodized by exponential decay function producing linear broadening of 2.0 Hz. After Fourier transform, automatic phasing was applied, and baselines were corrected by polynomial baseline correction.

Metabolite peaks were initially manually integrated and quantified relative to the TSP peak in each spectrum. Metabolites were quantified based on the ratio of the integral of the metabolite peak to the TSP peak previously adjusted to account for the ratio of metabolite and TSP protons giving rise to the signals chosen by integration (1:9)

The calculation equation of quantitative nuclear magnetic resonance (qNMR) for the analyte concentration is as follows:

C _(x-PHL)_ = [(Peak area of H_i_)/(Peak area of TSP)] × 9/1 × C_(TSP)_

This formula was also applied for the 3 peaks area, H-2, H-3 and H-4; from these 3 values, the average was taken as an experimental measure of the concentration of the targeted metabolite and the spread as the standard deviation of the measure. 

In order to confirm the accuracy of peak integration, peak deconvolution was also performed using the global spectral deconvolution tool (GSD) implemented in MestReNova software, allowing automatic peak integration of the deconvoluted peak. 

As an example for EC-PHL, we obtained

C_2(EC-PHL)_ = [(Peak area of H-2)/(Peak area of TSP)] × 9/1 × C_(__T*S*P__)_ = 9.60 mM

C_3(EC-PHL)_ = [(Peak area of H-3)/(Peak area of TSP)] × 9/1 × C_(__T*S*P__)_ = 9.59 mM

C_4(EC-PHL)_ = [(Peak area of H-4)/(Peak area of TSP)] × 9/1 × C_(__T*S*P__)_ = 9.70 mM

Average C_(EC-PHL)_ = (9.6 ± 0.2) mM.

The ^1^H-NMR spectra (400 MHz) of the isolated phloroglucinol adducts were also acquired in acetone-d6 at 300 K on the same NMR spectrometer, using a 5 mm BBI probe with 90° proton pulse length of 8.7 μs at a transmission power of -1db and equipped with pulsed gradient field utility. The chemical shift scale (δ) was calibrated in these measures on the residual signal of deuterated acetone at δ_H_ 2.05. ^1^H-NMR data of the isolated 4-phloroglucinols of epicatechin, epigallocatechin and epicatechin 3-*O*-gallate were found in fair agreement with previously reported NMR data [28,29].

EC-PhL: ^1^H-NMR (400 MHz, D_2_O): 6.92 (d, *J* = 2.0 Hz, 1H, H-2′), 6.84 (d, *J* = 8.2 Hz, 1H, H-8′), 6.79 (dd, *J* = 8.2, 2.0 Hz, 1H, H-6′), 6.06 (d, *J* = 2.0 Hz, 1H, H-8), 5.99 (d, *J* = 2.0 Hz, 1H, H-6), 5.94 (br s, 2H, H-3′′ + H-5′′), 5.17 (s, 1H, H-2), 4.40 (s, 1H, H-4), 3.92 (s, 1H, H-3). ^1^H-NMR (400 MHz, Acetone) δ 6.99 (d, *J* = 1.8 Hz, 1H, H-2′), 6.77 (d, *J* = 8.1 Hz, 1H, H-8′), 6.73 (dd, *J* = 8.2, 1.8 Hz, 1H, H-6′), 6.03 (d, *J* = 2.2 Hz, 1H, H-8), 5.92 (br s, 2H, H-3′′ + H-5′′), 5.06 (s, 1H, H-2), 4.61 (d, *J* = 1.6 Hz, 1H, H-4), 4.03 (s, 1H, H-3).

EGC-PhL: ^1^H-NMR (400 MHz, D_2_O) δ 6.51 (s, 2H, H-2′ + H-6′), 6.06 (br s, 1H, H-8), 5.96 (br s, 2H, H-3′′ + H-5′′), 5.93 (s, 1H, H-6), 5.11 (s, 1H, H-2), 4.39 (br s, Hz, 1H, H-4), 3.91 (s, 1H, H-3). ^1^H-NMR (400 MHz, Acetone) δ 6.47 (s, 2H, H-2′ + H-6′), 6.05 (d, *J* = 2.0 Hz, 1H, H-8), 6.02 (d, *J* = 2.0 Hz, 1H, H-6), 5.93 (s, 2H, H-3′′ + H-5′′), 4.99 (s, 1H, H-2), 4.60 (br s, 1H, H-4), 4.01 (s, 1H, H-3).

ECG-PhL: ^1^H-NMR (400 MHz, D_2_O) δ 6.87 (m, 3H, H-2′′′ + H-6′′′ + H-2′), 6.74 (m, 2H, H-5′ + H-6′), 6.13 (d, *J* = 2.0 Hz, 1H, H-8), 6.01 (d, *J* = 2.0 Hz, 1H, H-6), 5.94 (br s, 2H, H-3′′ + H-5′′), 5.42 (s, 1H, H-2), 5.26 (s, 1H, H-3), 4.49 (s, 1H, H-4). ^1^H-NMR (400 MHz, Acetone) δ 7.11 (s, 1H, H-2′), 7.08 (s, 2H, H-2′′′ + H-6′′′), 6.72 (m, 2H, H-5′ + H-6′), 6.09 (d, *J* = 2.3 Hz, 1H, H-8), 6.00 (d, *J* = 2.3 Hz, 1H, H-6), 5.97 (br s, 2H, H-3′′ + H-5′′), 5.45 (s, 1H, H-2), 5.14 (s, 1H, H-3), 4.61 (s, 1H, H-4).

### 4.7. Sample Preparation

Sample preparation for the measurements of the phenolic analytes was performed according to the method previously described (Gris et al. 2013) with slight modifications. Briefly, 10 mL of wine diluted 10 times with H_2_O was applied to a C18-SPE cartridge (1 g, Waters, Milford, MA, USA), previously activated with methanol (4 mL) and conditioned with H_2_O (10 mL). The cartridge was washed with 50 mL of H_2_O and then eluted with 40 mL of methanol into a 100 mL flask. The elute was evaporated under reduced pressure, reconstituted in 2 mL of methanol and finally filtered through 0.22 µm polytetrafluoroethylene (PTFE) filters into a 2 mL amber LC-MS certificated vial (Waters). For analysis before the phloroglucinol reaction, right before the analysis each purified sample was diluted 5 times with H_2_0/methanol 50/50. For the reaction, 100 µL of concentrated and purified wine sample reacted with 100 µL of the phloroglucinol reagent at 50 °C for 20 min and then combined with 1 mL of 40 mM aqueous sodium acetate to stop the reaction. The phloroglucinol reagent was a solution of 0.2 N HCl in methanol, containing 100 g/L of phloroglucinol and 20 g/L ascorbic acid. The final solution was filtered through a 0.22 µm PTFE filters into LC-MS certificated vials and immediately analyzed.

### 4.8. UHPLC-MS/MS Analysis

UHPLC-MS/MS analysis was performed on a Waters Acquity UPLC system (Milford, MA, USA). The method was based on methods previously described [30,38], with small modifications in order to add the new metabolites. Separation of phenolic compounds was achieved on a Waters Acquity HSS T3 column 1.8 μm, 150 mm × 2.1 mm (Milford, MA, USA) kept at 40 °C. The mobile phase was composed of eluent A (0.1% formic acid in water) and eluent B (0.1% formic acid in acetonitrile). The flow was set to 0.4 mL/min, and the gradient profile was: 0 min 5% B, from 0 to 3 min linear gradient to 20% B; from 3 to 4.30 min, isocratic gradient to 20% B; from 4.30 to 6 min linear gradient to 29% B, from 6.01 to 8 min isocratic gradient to 100% and from 8.01 to 13 min, re-equilibration to the initial conditions of 5% B. The injection volume was 2 μL for both sample and standard solutions. Each sample was analyzed in triplicate. After each injection, the needle was rinsed with 600 μL of a weak washing solution (water/methanol, 90:10) and 200 μL of a strong washing solution (methanol/water, 90:10). Samples were kept at 6 °C during the analysis.

Mass spectrometry detection was performed on a Waters Xevo TQMS (Milford, MA, USA) instrument equipped with an electrospray (ESI) source. Capillary voltage was 3.5 kV in positive mode and −2.7 kV in negative mode; the source was kept at 150 °C; desolvation temperature was 500 °C; cone gas flow, 50 L/h; and desolvation gas flow, 1000 L/h. Unit resolution was applied to each quadrupole. Flow injections of each individual metabolite were used to optimise the MRM conditions, automatically with Waters Intellistart software. All the MRM parameters can be found in Table 1, and the corresponding chromatograms can be found in Supplementary Figure S2.

Calibration curves were constructed for each standard at ten concentration levels, in a concentration range spanning >5 orders of magnitude, and by using the mobile phases (95% A and 5% B) for the dilutions. The limit of quantification (LOQ) for each compound was evaluated as the concentration at which the quantifier transition presented a signal-to-noise (S/N) ratio of >10, and for the limit of detection (LOD) a signal-to-noise (S/N) ratio of >3 (Table 1). The method/sample preparation precision was evaluated by preparing the same pooled wine sample 12 times, while instrumental reproducibility was measured by analyzing the same sample several times.

Each wine was analyzed both before and after the phloroglucinol reaction.

### 4.9. Data Processing and Statistical Treatment

Data processing was carried out using Waters MassLynx version 4.1 and TargetLynx software (Waters, Milford, MA, USA). One-way ANOVA with port-hoc Tukey’s HSD statistical analysis were performed using SPSS V19 (IBM Statistics), where statistical significance was considered as a hypothesis with a p-value less than 0.05. Further statistical analysis was performed using the MetaboAnalyst online platform version 4.0 (http://www.metaboanalyst.ca/ (accessed on 5 February 2021)) without normalization, missing value estimation and data transformation, using auto value scaling. 

For the calculation of the percentage of galloylation (%G) and mean degree of polymerization (mDP), the previously reported formulas were applied [30].

## 5. Conclusions

In conclusion, we developed a fast UHPLC-MS/MS protocol for the measurement of the monomeric and dimeric flavan-3-ols in wine and their products obtained after phloroglucinolysis. Using the three main flavanol phloroglucinol adducts as pure standards, and two injections for the sample (before and after the reaction), the results of the method are more robust and reliable, while the instrumental technology used offers sensitivity, selectivity and velocity. Moreover, we showed that the commercial wines made of Sagrantino belong to the group of wines with proanthocyanidins of high polymerization.

Degradative methods for the measurement of proanthocyanidins, like the phloroglucinolysis protocol(s), still have some drawbacks, producing partial results, and we need to resolve them in the future but they are still the best tool we have to obtain information about the oligomeric and polymeric condensed tannins.

## Figures and Tables

**Table 1:** UHPLC-MS/MS parameters.

Compound ^1^	RT (min)	Cone Voltage (V)	ESI Mode	Quantifier MRM (Collision Energy/V)	Qualifier MRM (Collision Energy/V)
B1	3.06	32	−	577.1 → 289.0 (26)	577.1 → 425.1 (16)
B2	3.67	30	−	577.1 → 289.0 (24)	577.1 → 425.1 (16)
C	3.50	32	−	289.0 → 203.0 (20)	289.0 → 123.0 (32)
EC	4.00	34	−	289.0 → 203.0 (20)	289.0 → 123.0 (30)
GC	2.56	32	−	305.0 → 124.9 (26)	305.0 → 179.0 (19)
EGC	3.15	32	−	305.0 → 124.9 (22)	305.0 → 179.0 (16)
ECG	5.25	32	−	441.0 → 289.0 (18)	441.0 → 169.0 (26)
CG	5.35	32	−	441.0 → 289.0 (18)	441.0 → 169.0 (27)
EC-Phl	3.02	40	−	413.3 → 125.0 (20)	413.3 → 261.1 (14)
EGC-Phl	2.28	25	−	429.4 → 125.0 (25)	429.4 → 177.0 (22)
ECG-Phl	3.88	28	−	565.5 → 125.0 (38)	565.5 → 395.2 (16)

^1^ B1: procyanidin B1, B2: procyanidin B2, C: catechin, EC: epicatechin, EGC: epigallocatechin, ECG: epicatechin 3-*O*-gallate, catechin 3-*O*-gallate, Phl: phloroglucinol adduct.

**Table 2:** Calibration parameters of the UHPLC-MS/MS method.

Compound ^1^	LOD–LOQ ^2^	Range (mg/L)	Calibration Curve	Correlation Coefficient
B1	0.039–0.132	0.006–16.20	y = 826.07x + 0.96	r = 0.987
B2	0.028–0.078	0.013–32.42	y = 1498.46x − 9.94	r = 0.986
C	0.063–0.387	0.020–101.85	y = 930.83x + 70.44	r = 0.994
EC	0.069–0.386	0.069–68.70	y = 888.96x + 116.86	r = 0.982
GC	0.027–0.076	0.019–46.75	y = 2023.71x − 12.14	r = 0.985
EGC	0.024–0.068	0.011–28.12	y = 2345.22x − 12.26	r = 0.987
ECG	0.012–0.037	0.011–27.50	y = 7623.12x − 7.09	r = 0.998
CG	0.032–0.078	0.025–63.12	y = 1793.95x − 21.87	r = 0.985
EC-Phl	0.322–0.442	0.322–321.6	y = 1202.57x + 92.00	r = 0.990
EGC-Phl	0.153–0.198	0.153–153.0	y = 861.83x − 77.76	r = 0.985
ECG-Phl	0.083–0.141	0.083–432.2	y = 716.06x + 24.53	r = 0.991

^1^ B1: procyanidin B1, B2: procyanidin B2, C: catechin, EC: epicatechin, EGC: epigallocatechin, ECG: epicatechin 3-*O*-gallate, catechin 3-*O*-gallate, Phl: phloroglucinol adduct. ^2^ LOD: Limit of detection, LOQ: Limit of quantification.

## Data Availability

The data presented in this study are available in the Supplementary Materials, and raw files are available on request from the corresponding author.

## Supplementary Materials

The following are available online, Table S1: Wine samples data, meta-data, and quantitative analysis. Table S2: Anova Statistical analysis. Figure S1: NMR spectra of the flavanol phloroglucinol adducts, epicatechin 4-phloroglucinol (EP-Phl), epicatechin-gallate 4-phloroglucinol (ECG-Phl) and epigallocatechin 4-phloroglucinol (EGC-Phl). Figure S2: LC-MS chromatograms of the flavan-3-ols with and without the phloroglucinol adduct.

## Sample Availability

Samples of the synthesized compounds are available from the authors.
