# Supplementary material for: Improving the Phloroglucinolysis Protocol and Characterization of Sagrantino Wines Proanthocyanidins
Source: Molecules. 2021 Feb 19;26(4):1087. doi: 10.3390/molecules26041087 (PMC7922431; doi:10.3390/molecules26041087)
Supplement: Supplementary file 1 [file molecules-26-01087-s001.zip › molecules-1040702_Supplementary_materials.pdf]

Supplementary materials

Improving the Phloroglucinolysis protocol and characterization of Sagrantino wines proanthocyanidins

Panagiotis Arapitsas [1], Daniele Perenzoni [1] Graziano Guella [2] and Fulvio Mattivi [1,3,*]

[1] Department of Food Quality and Nutrition, Research and Innovation Centre, Fondazione Edmund Mach, San Michele all'Adige (TN), Italy; panagiotis.arapitsas@fmach.it daniele.perenzoni@fmach.it

[2] Department of Physics, University of Trento, Via Sommarive 14, 38123 Povo Trento, Italy; graziano.guella@unitn.it

[3] Department of Cellular, Computational and Integrative Biology - CIBIO, University of Trento, San Michele all'Adige, Italy; fulvio.mattivi@unitn.it

[*] Correspondence: fulvio.mattivi@unitn.it Tel.: +39 0461615259

Supplementary Material Description

1. Supplementary Figure S1. ^{1}H-NMR spectra of the flavanol phloroglucinol adducts.
2. Supplementary Figure S2. LC-MS chromatograms of the flavanols with and without the phloroglucinol adduct.
3. Supplementary Table S1. Wine samples data, meta-data, and quantitative analysis. (Separate excel file)
4. Supplementary Table S2. Anova Statistical analysis. (Separate excel file)

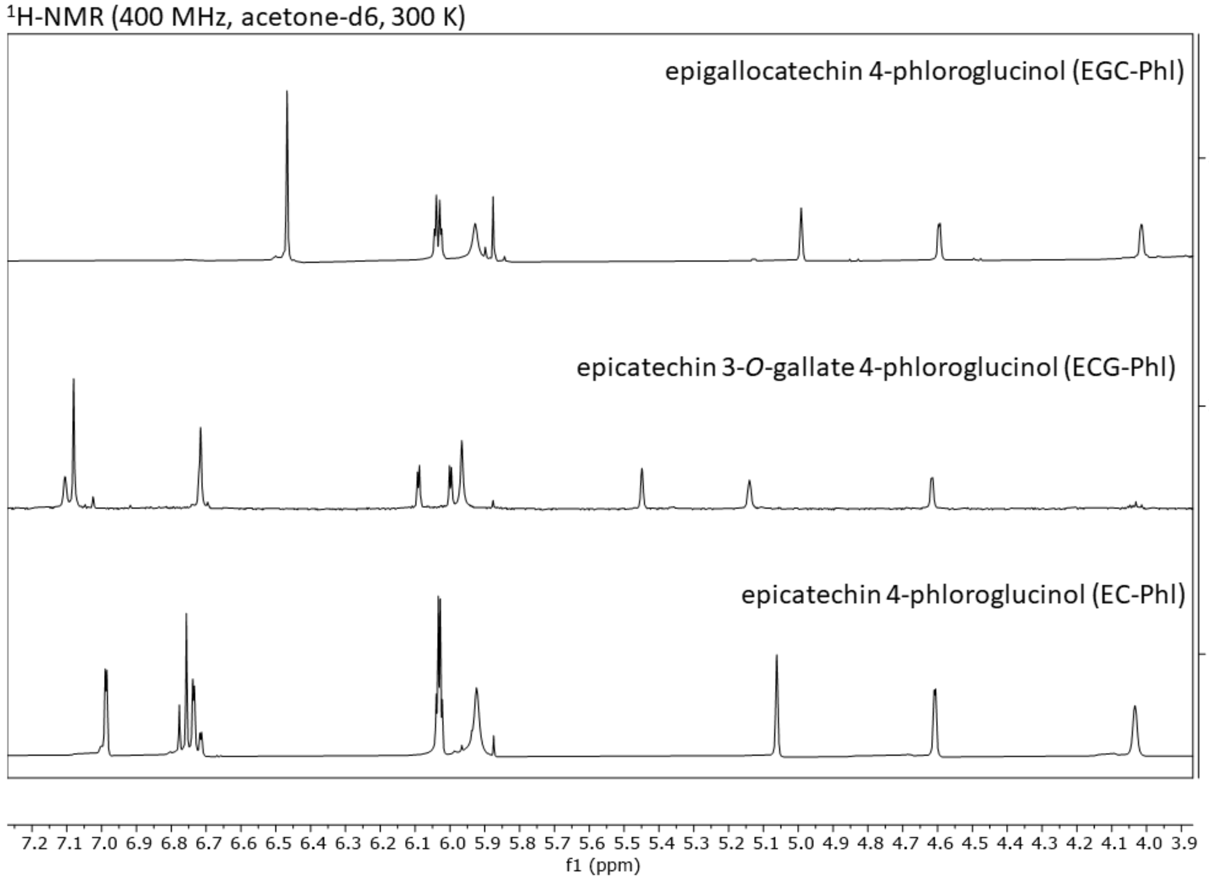

Supplementary Figure S1. ^{1}H-NMR spectra of the flavanol phloroglucinol adducts.

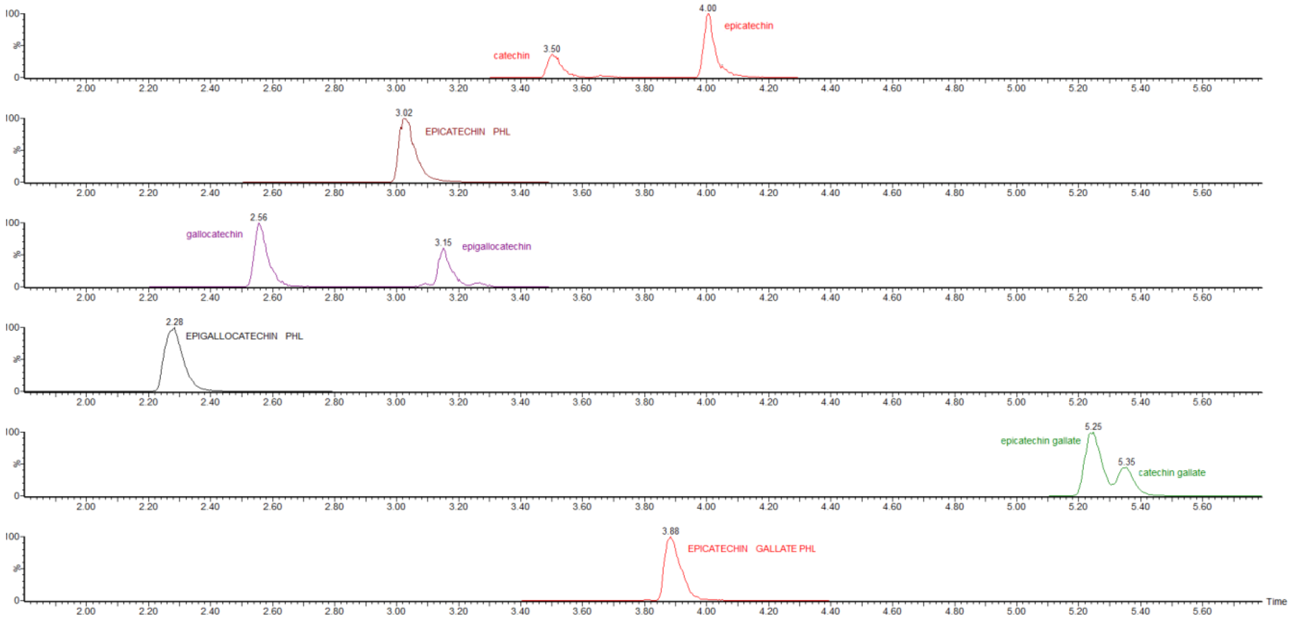

Supplementary Figure S2. LC-MS chromatograms of the flavanols with and without the phloroglucinol adduct.
